# Silymarin exerts antipsoriatic effects against imiquimod-induced psoriasis in mice via NF-kB/TLR4 signaling pathway

**DOI:** 10.22038/ijbms.2025.87874.18981

**Published:** 2025

**Authors:** Behnaz Azimi, Amir Kiani, Tayebeh Noori, Antoni Sureda, Samira Shirooie

**Affiliations:** 1 Student Research Committee, Faculty of Pharmacy, Kermanshah University of Medical Sciences, Kermanshah, Iran; 2 Pharmaceutical Sciences Research Center, Health Institute, Kermanshah University of Medical Sciences, Kermanshah, Iran; 3 Medical Biology Research Center, Health Technology Institute, Kermanshah University of Medical Sciences, Kermanshah, Iran; 4 Research Group on Community Nutrition and Oxidative Stress (NUCOX) and Health Research Institute of Balearic Islands (IdISBa), University; 5 of Balearic Islands-IUNICS, Palma de Mallorca E-07122, Balearic Islands, Spain; 6 CIBER Fisiopatología de la Obesidad y Nutrición (CIBEROBN), Instituto de Salud Carlos III (ISCIII), 28029 Madrid, Spain

**Keywords:** Imiquimodm, Inflammation, Male mice, Psoriasis, Silymarin

## Abstract

**Objective(s):**

Psoriasis is an autoimmune disease that mainly affects the skin and joints, which is mediated via T-cells. Several factors contribute to its pathogenesis, including genetic and environmental triggers, as well as intrinsic immune processes that lead to an autoimmune response. Silymarin, a flavonoid complex extracted from *Silybum marianum*, exhibits anti-inflammatory, immunostimulatory, and anti-oxidant properties, rendering it a viable candidate for treating psoriasis.

This study aimed to investigate the effect of silymarin on imiquimod (IMQ) induced psoriasis-like skin lesions in male mice applied as a cream for seven consecutive days (1 mg per mouse).

**Materials and Methods:**

Thirty-five male mice were assigned to seven groups (n=5 per group): (I) control group, (II) IMQ group, (III-V) oral silymarin groups (30, 60, and 120 mg/kg), (VI) topical betamethasone group, and (VII) topical silymarin 2% group.

**Results:**

Silymarin, both orally and topically, significantly reduces erythema, thickness, and scaling induced by IMQ after seven days of treatment. The treatment also reversed the increase in spleen weight/body weight ratio. Immunofluorescence analysis revealed that silymarin reduced the expression of nuclear factor κB (NF-κB) (*P*<0.01) and toll-like receptor 4 (TLR4) (*P*<0.01) compared to the IMQ group.

**Conclusion:**

These findings suggest that silymarin effectively alleviates psoriasis lesions by reducing inflammation and modulating the TLR4/ NF-κB signaling pathway.

## Introduction

Psoriasis is a common, chronic, immune-mediated disease that primarily affects the skin and joints (1), impacting 2–4% of the adult population and 0.1–1% of children (2). Many factors contribute to the development of psoriasis, including genetic tendency, infections, psychological distress, low vitamin D levels, and certain medications, such as beta blockers, lithium, and antimalarial medicines, which play significant roles (3). The clinical finding of psoriasis is primarily reliant on the Psoriasis Area and Severity Index (PASI) score due to the lack of effective diagnostic methods for this condition (4). It is characterized by the presence of red, scaly patches that may develop on various parts of the body, resulting in considerable physical and emotional distress for those affected (5). Psoriasis plaques are characterized by a congealed epidermis with whitish scale, erythema, and the recruitment of inflammatory cells (6). The pathogenesis of psoriasis can be clarified by the dysregulation of immunological cell function, which leads to uncontrolled keratinocyte proliferation and dysfunctional differentiation (7). This dysregulation may occur in immune-related cells, including dendritic cells (DCs) and macrophages, as well as in Toll-like receptors and cytokines such as interferon-α (IFN-α), tumor necrosis factor-α (TNF-α), interleukin (IL) 23, and IL-17 (8). TLRs are innate immune receptors that can activate innate and adaptive immune pathways by detecting pathogen-associated molecular patterns (PAMPs) and responding to them by triggering a wide array of downstream signaling pathways, resulting in distinct immune profiles (9). A mammalian homolog of the Toll receptor, now known as TLR4, has been demonstrated to induce the expression of genes related to inflammatory responses (10).

Psoriasis inhibits the function of TLR4 on dendritic cells, leading to their dysfunction, reduced secretion of anti-inflammatory cytokines, and a decrease in hypersensitivity reactions and inflammation (11). The elevation of TLR4 gene expression in psoriasis and its positive correlation with disease severity indicate that this receptor dysregulation has a prominent role in the pathogenesis of psoriasis and targeting and inhibiting TLR4 have an effective role in treatment or inhibiting TLR4 may have an effective role in the treatment of this condition(12). The nuclear factor κB (NF-κB) belongs to a family of transcription factors that regulate a vast number of biological responses (13). NF-κB is an essential nuclear transcription factor in immune cell and keratinocyte biology and plays an important role in the inflammation (14) It has been demonstrated NF-κB translocation in lymphocytes within psoriatic skin triggers its subsequent translocation in the epidermis and basal cells which is responsible for two of the main features of psoriasis, epidermal hyperplasia, and inflammation (15) In addition, the activation of NF-κB signaling by inducing the expression of keratins 6 and 16 in keratinocytes, can lead to acanthosis and decreased turnover time in the epidermis (16). Therefore, targeting or inhibition of NF-κB should be considered for psoriasis treatment (17).

Imiquimod (IMQ) binds to the TLR7/8 receptor and is a potent immune activator, with FDA approval for the topical treatment of genital/perianal warts caused by human papillomavirus (HPV) type 18. Furthermore, topical application of IMQ has been found to persuade a dermatitis in mice thoroughly like human psoriasis (19) IMQ can activate the production of downstream factors such as TNF -α, IL -1β, IL -6, and IL -23 by binding with epidermal plasma -like dendritic cells and Toll -like receptor (TLR) -7 in macrophages, mimicking inflammatory changes in psoriasis (20). It has been demonstrated that the daily application of IMQ induces symptoms of psoriasis, such as scaling, erythema, and epidermal hyperplasia (21).

Currently, there is no cure for psoriasis; however, suppressive therapy (22) is available, including topical, systemic, phototherapy, combination therapy, herbal therapy, and novel molecules (23). Psoriasis treatment is recommended based on the severity of the disease (24). Topical therapy is the first-line treatment in the management of mild-to-moderate psoriasis (25) and, due to its efficacy and safety profile, plays a crucial role in psoriasis treatment (26). Still, studies have shown that medication adherence rates are lower for topical treatment than for systemic treatment. Poor medication adherence is associated with unfavorable treatment outcomes, an increased risk of developing associated diseases, and the inefficient use of healthcare resources (27).

Research indicates that medicinal plants serve as a rich source of various therapeutic compounds and have been used globally for many years to treat a wide range of health issues (28). Flavonoids represent the most prevalent and extensively distributed category of plant compounds, found in nearly all parts of plants, which can be categorized into various subfamilies, including flavanols, flavones, flavanones, flavonols, and isoflavones (29). Research has consistently demonstrated that flavonoids exert antipsoriatic effects by modulating several molecular mechanisms related to inflammation, cytokine production, and keratinocyte proliferation, among other processes (30, 31).

Silymarin is a plant-derived combination of polyphenolic flavonoids obtained from the dried seeds and fruits of *Silybum marianum *(28), which contains around 50% silybin, 20% silychristin, 10% silydianin, 5% isosilybin, and between 10 and 30% of an anonymous organic polymer portion formed from these compounds (32). Silybin is the most functionally active compound among them (33). Both silymarin and silibinin display anti-oxidant, anti-inflammatory, immunomodulatory, and hepatoprotective properties (34). These protective effects have been demonstrated in nephrotoxicity, viral hepatitis, cancer, fertilization, neurotoxicity, depression, and lung diseases (35). Studies have shown that silymarin, by interfering with NF-κB–controlled transcriptional processes involved in tissue injury and cellular proliferation, can support processes that induce cell growth arrest and apoptosis, while inhibiting these processes and promoting abnormal cell accumulation (36). In addition, silymarin has been shown to decrease interleukin (IL)-6, IL-1β, and IL-12β, as well as tumor necrosis factor α (TNF-α), thereby modulating the immune system response (37). Additionally, silymarin can help reduce inflammation and regulate lipid uptake, synthesis, and oxidation by influencing microbial metabolites that impact the TLR4/NF-κB and FXR signaling pathways.

 Oral administration of silymarin has relatively low Absorption (39). However, a plasma level of 500 mg/l (as silibinin) has been reported 90 minutes after oral administration of 200 mg/kg of silymarin in mice (40). The peak plasma concentration is achieved in 6 to 8 hr (41), and its elimination half-life ranges from 6 to 8 hr, with excretion primarily occurring through bile and, to a lesser extent, through urine (42). Silymarin has been demonstrated to be safe and non-toxic in both animals and humans (39), with LD50 values reported as 400 mg/kg b.w. in mice, 385 mg/kg b.w. in rats, and 140 mg/kg b.w. in rabbits and dogs following intravenous infusion (43)

This study aimed to formulate an Eucerin-based ointment and examine the therapeutic effects of both topical and oral silymarin in a psoriasis mouse model induced by imiquimod. The research involved assessing the Psoriasis Area Severity Index (PASI), the spleen-to-body weight ratio, and the expression levels of TLR4 and NF-κB using immunohistochemical techniques in a mouse model.

## Materials and Methods

### Chemicals

Silymarin (SILY) and imiquimod propionate cream patches were obtained from Sigma Aldrich. Betamethasone 0.1% ointment and Eucerin were purchased from Abu Raihan Pharmaceutical Company. Monoclonal antibodies against mouse TLR4 and NF-kappa p65 ((F-6): sc-8008) were used for immunohistochemical assays.

### Animals

In the present study, 35 male mice weighing between 160 and 180 g were used. Mice were purchased from Elm Bavaran Aftab Company. All animals were housed under identical conditions, with a 12-hr light/dark cycle, a temperature of 25 °C, and humidity of 50±5%. The animal had free access to tap water and food. 

### Preparation of topical ointment containing silymarin (2%w/w)

Based on the solubility of silymarin in Eucerin, a 2% silymarin ointment was prepared by dissolving 500 mg of silymarin in 10 g of Eucerin (44).

### Induction of imiquimod-psoriasis and silymarin treatment

One day prior to study, the dorsal skin of each mouse was shaved using hair elimination cream. Then, the mice were randomly divided into seven different groups (n = 5 per group): 1) control group: received a standard diet daily for 7 days; 2) IMQ group: IMQ cream (1 mg per mouse) was applied evenly every day for 7 days; 3) oral silymarin 30 mg/kg group: received silymarin (30 mg/kg) dissolved in water; 4) oral silymarin 60 mg/kg group; 5) oral silymarin 120 mg/kg group; 6) topical betamethasone group:, betamethasone (0.1%) was applied to the shaved area for 7 days; 7) topical silymarin group: silymarin ointment (2%) was applied to the shaved area for 7 days (45).

 In topical treatment groups, IMQ cream was applied to the shaved area 30 min after the topical treatment. The doses of silymarin and IMQ were selected based on a previous study (46, 47). On the last day, animals were euthanized with an intraperitoneal injection of 10 mg/kg xylazine and 50 mg/kg ketamine. Skin and spleen were collected for additional analysis.

### Evaluating the score of the psoriasis area severity index (PASI)

The PASI score is the most widely used index measure of psoriasis severity, assessing lesion area, erythema, scaliness, and skin thickness (48). To determine the PASI score, mice were examined throughout the 7-day period. Erythema, scaling, and thickening were recorded on a scale from 0 to 4 as follows: 0, none; 1, slight; 2, moderate; 3, marked; 4, severe. The cumulative score (erythema + scaling + thickening) was considered equivalent to the PASI score to assess the severity of inflammation (49).

### Spleen weight to body weight index

Imiquimod increases spleen weight and its cell count by inducing systemic inflammation (50). Splenomegaly is a symptom of a generalized inflammatory response, and an increased spleen-to-body proportion indicates that the spleen is populated with more immune cells, suggesting a heightened immune response (51). On the last day of the study, after assessing the PASI Score and recording the animals’ weights, they were sacrificed. The spleens were carefully dissected, cleaned, and weighed. Then the proportion of spleen weight to body weight (organ index) was reported in mg/g.

### TLR4 and p65 NF-κB immunostaining

To evaluate the expression changes of TLR4 and NF-κB through immunostaining, 5-micrometer slices of formalin-preserved skin samples were placed on slides coated with saline. Then, they were deparaffinized and hydrated with ethanol and xylene (graded series), and washed with TBS plus 0.03% Triton X-100 with gentle agitation. After retrieval in citrate buffer (pH 6.2), the slides were blocked in 10% normal serum or 1% BSA in TBS for two hours at room temperature. The slides were incubated at 2–8 °C for 24 hr with normal donkey serum (10%) containing a rabbit monoclonal NF-κB antibody (phospho S40, 1:100 dilution, Abcam, USA), or a rabbit polyclonal TLR4 alpha antibody (Ser32/S36 phospho, 1:100 dilution, Elabscience, USA). After washing the slides three times for 5 min in TBS plus 0.03% Triton X-100, they were incubated with a goat anti-rabbit IgG (H+L) (FITC) antibody (orb688925) at a dilution of 1:150 for 1.5 hr at 37 °C. In the next step, after washing the slides with TBS plus 0.03% Triton X-100 three times and counterstaining with DAPI for 15 min at room temperature, they were dehydrated with ethanol and xylene (graded series). In the final step, after mounting and placing the coverslip, the slides were assessed with a fluorescence microscope (Olympus BX50) and camera (Olympus DP72) (52, 53).

### Statistical analysis 

Statistical analysis was completed using Prism 9 software. The results are reported as the mean ± SEM. One-way ANOVA with *post hoc* Tukey test (for TLR4, p65 NF-kB, and spleen/body weight analysis) and two-way ANOVA repeated measure (for erythema, scaling, thickness, and PSAI factors) with Bonferroni *post hoc* test were used to compare the mean differences between different groups, and *P*<0.05 was considered statistically significant.

## Results

### Effect of silymarin on IMQ-induced psoriatic-like skin inflammation

**Figure 1:**
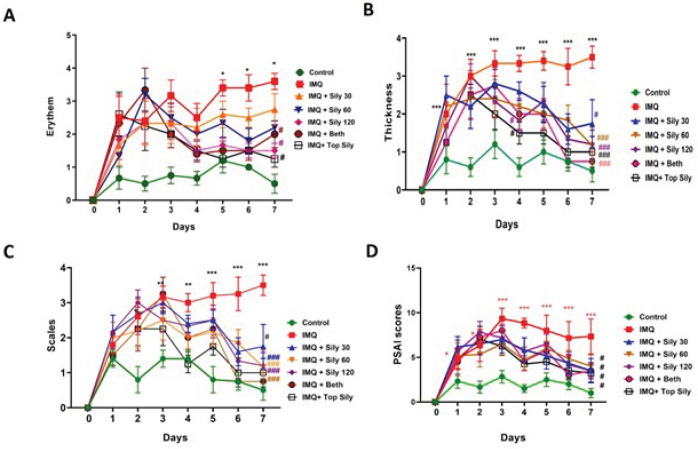
Results of topical and oral silymarin effect on (A) erythema; (B) thickness; (C) scales; (D) PASI score of each group following IMQ-induced psoriasis in mice

**Figure 2.**
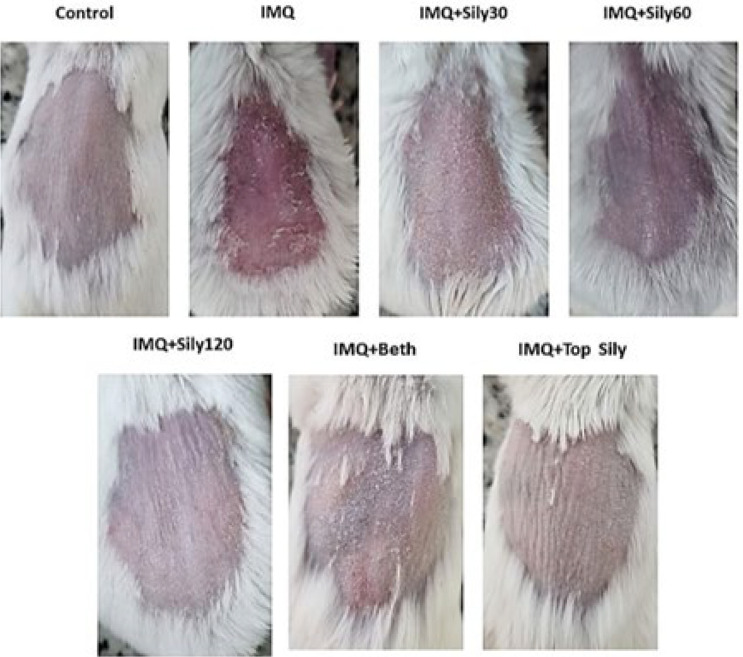
Clinical phenotypic characteristics changes after 7 days in mice

The fluctuations in clinical phenotypic characteristics, including erythema, scaling, and thickness, were evaluated to measure the healing effect of silymarin on psoriasis induced by IMQ (Figure 1A-D and Figure 2). IMQ significantly elevated these factors compared to the control (*P*<0.001). In contrast, treatment with topical silymarin (2%) and oral silymarin at doses of 60 and 120 mg/kg for seven consecutive days, similar to topical betamethasone ointment, significantly decreased the thickness, scaling, erythema (*P*<0.001), and PSAI (*P*<0.05) compared to the IMQ-only group.

### Effect of silymarin on spleen size in mice with IMQ-induced psoriasis

**Figure 3:**
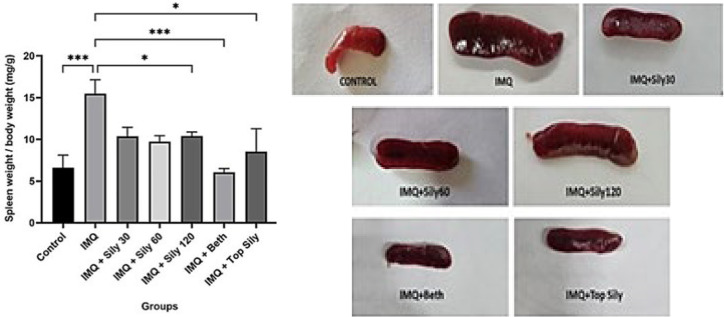
Oral and topical silymarin effect on spleen/body weight and spleen morphology of each group following IMQ-induced psoriasis in mice

At the end of the study, the IMQ group demonstrated a significantly larger spleen/body weight ratio compared to the control group (*P*<0.001), suggesting splenomegaly in mice, which indicates inflammation. In contrast, the topical and oral silymarin groups, at a dose of 120 mg/kg (*P*<0.05) and the betamethasone group (*P*<0.001), showed a considerable decrease in the spleen/body weight proportion compared to the IMQ group (Figure 3).

### Effect of oral and topical silymarin on TLR4 and p65 NF-κB protein expression 

**Figure 4:**
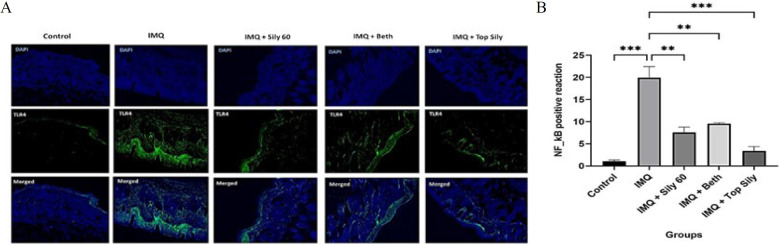
(A) TLR4 fluorescence immunostaining of the skin (× 40) of the mice, B) the optical intensity of IHC assay results using image

**Figure 5:**
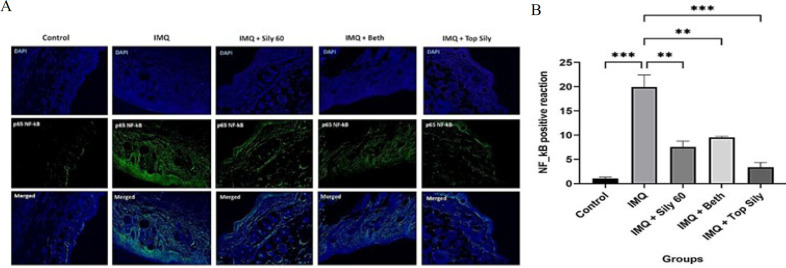
(A) p65 NF-κB fluorescence immunohistochemistry staining (IHC) (× 40) of the skin of the mice, (B) Optical intensity of IHC assay results using image

The levels of TLR4 and NF-κB proteins were measured by fluorescence immunostaining to assess the effects of IMQ, silymarin 2% ointment, betamethasone ointment, and oral silymarin (60 mg/kg). The results are presented in Figures 4 and 5. The expression level of TLR4 was meaningfully increased in the IMQ group, compared to the control group (*P*<0.01). However, topical and oral administration of silymarin (*P*<0.01) and topical betamethasone (*P*<0.05) significantly rectified the elevated TLR4 levels observed in the IMQ group. Furthermore, via IMQ administration, NF-κB protein levels were significantly higher than those in the control group (*P*<0.001). Conversely, treatment with silymarin, both orally (*P*<0.01) and topically (*P*<0.001), and topical betamethasone (*P*<0.05) significantly decreased the increased levels of NF-κB compared to the IMQ group.

## Discussion

In our research, the IMQ application for a week resulted in elevated spleen weight, changes in body weight, erythema, scaling, skin thickness, and a PASI score, which is consistent with prior studies (21). In contrast, topical and oral silymarin showed therapeutic effects in the skin, reducing the spleen-to-body weight index, erythema, scaling, skin thickness, and PASI. Many studies have indicated that silymarin possesses anti-oxidant, anti-inflammatory, and immunomodulatory properties (54) and reduces autoimmune, allergic, preeclampsia, cancer, and immune-mediated liver diseases (55). Moreover, it has been confirmed that the cell cycle in normal epidermis is nearly 311 hr, which is reduced to 36 hr in psoriasis lesions (56)**. **Singh and colleagues reported that silymarin, by modulating cell-cycle regulators and checkpoints, can inhibit proliferation and induce growth arrest in the G0-G1 and G2-M phases of the cell cycle (57). In the present study, local and oral treatment of silymarin resulted in a reduction in inflammation and skin lesions through the downregulation of NF-κB and TLR4 expression. In many studies, psoriasis is characterized by elevated levels of phosphorylated NF-κB, which serves as an important mediator in the pathogenesis of this condition (58). Al-Rasheed and his colleagues’ study on the effect of silymarin against hepatotoxicity induced by CCl_4 _showed that silymarin can significantly reduce the level of NF-κB compared to the control group and the group that only received CCl_4_ (59). Furthermore, Altindağ and his colleagues’ study on the effect of silymarin against cisplatin-induced nephrotoxicity in rats indicated that NF-κB and TNF-α levels are significantly reduced in the silymarin-treated group compared to the cisplatin-induced nephrotoxicity group (60).

TLR4, which can be activated by LPS (61), plays a substantial role in regulating pro-inflammatory gene expression through the activation of intracellular signaling pathways, including NF-κB (62). The activation of TLR4 initiates a cascade that leads to the translocation of NF-κB to the nucleus and enhances the expression of pro-inflammatory cytokines, including IL-1β, IL-6, and TNF-α. Targeting these molecules and their upstream signaling pathways presents a promising therapeutic strategy for addressing inflammatory diseases, including psoriasis (63). Furthermore, silymarin has been shown to mitigate inflammation and modulate lipid uptake, synthesis, and oxidation by regulating microbial metabolites, thereby affecting the TLR4/NF-κB and FXR signaling pathways (38).

 Fluorescence immunostaining results demonstrated that IMQ significantly elevated the protein expression of TLR4 and p65 NF-κB in mice. Conversely, topical and oral administration of silymarin led to a reduction in inflammation by lowering the levels of TLR4 and p65 NF-κB. Moreover, clinical phenotypic characteristics demonstrated that silymarin diminished dermal fibrosis, reduced epidermal thickness, and decreased infiltration of inflammatory cells induced by IMQ in animals, suggesting a therapeutic effect of silymarin in managing psoriasis skin lesions. Overall, the advantages of this study include the utilization of a well-established imiquimod-induced psoriasis model and a thorough investigation of the mechanism of silymarin action via the NF-κB/TLR4 signaling pathway. The findings are supported by comprehensive and rigorous laboratory analyses, demonstrating the therapeutic potential of silymarin as a natural agent. The disadvantages include the relatively small sample size, which may pose some limitations in statistical power. Additionally, due to biological differences, animal models cannot fully replicate all aspects of human psoriasis. This study primarily focused on evaluating the efficacy of silymarin, and direct comparisons with standard psoriasis treatments are suggested for future research.

*P<0.05, **P<0.01, *** *P*<0.001 comparing with control group. #*P*<0.05, ## *P*<0.01, ### *P*<0.001 comparing with IMQ group, according to two-way ANOVA repeated measure. Data are presented as mean ± SD. PASI: Psoriasis area severity index; IMQ: Imiquimod

Control group: receiving normal water and food; IMQ, psoriasis group receiving daily topical applications of IMQ cream (5%) on the shaved back skin for seven consecutive days; IMQ+Sily30, 60 and 120 receiving a daily oral dose of silymarin 30 mg/kg, 60 mg/kg and 120 mg/kg, respectively after receiving IMQ for 1 week; IMQ+Bet group received topical betamethasone after receiving IMQ for 1 week: IMQ+Top Sily received topical silymarin ointment after receiving IMQ for 1 week

IMQ: Imiquimod; Sily: Silymarin; Bet: Betamethasone

*P<0.05, **P<0.01, *** *P*<0.001. Data are presented as mean ± SD. IMQ: Imiquimod, Sily: Silymarin, Beth: Betamethasone

*P<0.05, **P<0.01, ***P<0.001. IMQ: Imiquimod, Sily: Silymarin, Beth: Betamethasone

*P<0.05, **P<0.01, ***P<0.001. IMQ: Imiquimod, Sily: Silymarin, Beth: Betamethasone

## Conclusion

Psoriasis is a prevalent chronic inflammatory skin condition associated with immune system dysfunction, characterized by the rapid proliferation of skin cells. The topical application of IMQ is a widely used method for inducing psoriasis in animal models, resulting in skin alterations that mimic psoriatic lesions. In this study, the application of silymarin, both topically and orally, demonstrated improvements in skin thickness, erythema, and scaling in mice. Additionally, silymarin effectively moderated the ratio of spleen weight to body weight, addressing one of the side effects commonly associated with psoriasis. The therapeutic effects of silymarin primarily stem from its anti-inflammatory properties, which are mediated through the downregulation of the NF-ΚB pathway and the inhibition of TLR4 activation. These results suggest that topical and oral silymarin may serve as a viable treatment option for alleviating psoriasis lesions and their associated complications.

## Declaration

We have not used any AI tools or technologies to prepare this manuscript.

## References

[1] Boehncke WH, Schön MP (2015). Psoriasis. Lancet..

[2] Schäkel K, Schön MP, Ghoreschi K (2016). Pathogenesis of psoriasis. Hautarzt.

[3] Branisteanu DE, Cojocaru C, Diaconu R, Porumb EA, Alexa AI, Nicolescu AC (2022). Update on the etiopathogenesis of psoriasis (Review). Exp Ther Med.

[4] Jin Z, Huang Q, Peng J, Liu Z, Hu R, Wu J (2023). MiR-125a-3p alleviates hyperproliferation of keratinocytes and psoriasis-like inflammation by targeting TLR4/NF-κB pathway. Postepy Dermatol Alergol.

[5] Lee HJ, Kim M (2023). Challenges and future trends in the treatment of psoriasis. Int J Mol Sci.

[6] Rujimongkon K, Ampawong S, Reamtong O, Buaban T, Aramwit P (2021). The therapeutic effects of Bombyx mori sericin on rat skin psoriasis through modulated epidermal immunity and attenuated cell proliferation. J Tradit Complement Med.

[7] Rendon A, Schäkel K (2019). Psoriasis pathogenesis and treatment. Int J Mol Sci.

[8] Tokuyama M, Mabuchi T (2020). New treatment addressing the pathogenesis of psoriasis. Int J Mol Sci.

[9] Kawai T, Akira S (2007). TLR signaling. Semin Immunol.

[10] Takeda K, Akira S (2005). Toll-like receptors in innate immunity. Int J Mol Sci.

[11] Panzer R, Blobel C, Fölster-Holst R, Proksch E (2014). TLR 2 and TLR 4 expression in atopic dermatitis, contact dermatitis and psoriasis. Exp Dermatol.

[12] Shehata W, Hammam M, Seif D, Elsayed S, Maqsoud E, El-Hefnawy S (2022). Role of Toll-like receptor 4 gene expression in psoriasis and its relation to disease activity. Menoufia Med J.

[13] Dolcet X, Llobet D, Pallares J, Matias-Guiu X (2005). NF-kB in development and progression of human cancer. Virchows Arch.

[14] Gaptulbarova KA, Tsyganov MM, Pevzner AM, Ibragimova MK, Litviakov NV (2020). NF-kB as a potential prognostic marker and a candidate for targeted therapy of cancer. Exp Oncol.

[15] Moorchung N, Kulaar JS, Chatterjee M, Vasudevan B, Tripathi T, Dutta V (2014). Role of NF-κB in the pathogenesis of psoriasis elucidated by its staining in skin biopsy specimens. Int J Dermatol.

[16] Ogawa E, Sato Y, Minagawa A, Okuyama R (2018). Pathogenesis of psoriasis and development of treatment. J Dermatol.

[17] Goldminz AM, Au SC, Kim N, Gottlieb AB, Lizzul PF (2013). NF-κB: An essential transcription factor in psoriasis. J Dermatol Sci.

[18] van der Fits L, Mourits S, Voerman JSA, Kant M, Boon L, Laman JD (2009). Imiquimod-induced psoriasis-like skin inflammation in mice is mediated via the IL-23/IL-17 axis1. J Immunol.

[19] Gao J, Chen F, Fang H, Mi J, Qi Q, Yang M (2020). Daphnetin inhibits proliferation and inflammatory response in human HaCaT keratinocytes and ameliorates imiquimod-induced psoriasis-like skin lesion in mice. Biol Res.

[20] Greaves MW, Weinstein GD (1995). Treatment of psoriasis. NEJM.

[21] Rahman M, Alam K, Ahmad MZ, Gupta G, Afzal M, Akhter S (2012). Classical to current approach for treatment of psoriasis: A review. Endocr Metab Immune Disord Drug Targets.

[22] Rapalli VK, Sharma S, Roy A, Singhvi G (2021). Design and dermatokinetic evaluation of Apremilast loaded nanostructured lipid carriers embedded gel for topical delivery: A potential approach for improved permeation and prolong skin deposition. Colloids Surf. B Biointerfaces.

[23] Mitra A, Wu Y (2010). Topical delivery for the treatment of psoriasis. Expert Opin Drug Deliv.

[24] Feldman SR, Horn EJ, Balkrishnan R, Basra MK, Finlay AY, McCoy D (2008). Psoriasis: Improving adherence to topical therapy. J Am Acad Dermatol.

[25] Zschocke I, Mrowietz U, Karakasili E, Reich K (2014). Non-adherence and measures to improve adherence in the topical treatment of psoriasis. J Eur Acad Dermatol Venereol.

[26] Wadhwa K, Pahwa R, Kumar M, Kumar S, Sharma PC, Singh G (2022). Mechanistic insights into the pharmacological significance of silymarin. Molecules.

[27] Dobrzynska M, Napierala M, Florek E (2020). Flavonoid nanoparticles: A promising approach for cancer therapy. Biomolecules.

[28] Ebrahimi A, Mehrabi M, Miraghaee SS, Mohammadi P, Fatehi Kafash F, Delfani M (2024). Flavonoid compounds and their synergistic effects: Promising approaches for the prevention and treatment of psoriasis with emphasis on keratinocytes – A systematic and mechanistic review. Int Immunopharmacol.

[29] Alalaiwe A, Lin C-F, Hsiao C-Y, Chen E-L, Lin C-Y, Lien W-C (2020). Development of flavanone and its derivatives as topical agents against psoriasis: The prediction of therapeutic efficiency through skin permeation evaluation and cell-based assay. Int J Pharm.

[30] Zholobenko A, Modriansky M (2014). Silymarin and its constituents in cardiac preconditioning. Fitoterapia..

[31] Koltai T, Fliegel L (2022). Role of silymarin in cancer treatment: Facts, hypotheses, and questions. J Evid Based Integr Med.

[32] Chambers CS, Holečková V, Petrásková L, Biedermann D, Valentová K, Buchta M (2017). The silymarin composition… and why does it matter???. Food Res Int.

[33] Karimi G, Vahabzadeh M, Lari P, Rashedinia M, Moshiri M (2011). “Silymarin”, a promising pharmacological agent for treatment of diseases. Iran J Basic Med Sci.

[34] Comelli MC, Mengs U, Schneider C, Prosdocimi M (2007). Toward the definition of the mechanism of action of silymarin: Activities related to cellular protection from toxic damage induced by chemotherapy. Integr Cancer Ther.

[35] Rostamian S, Heidari-Soureshjani S, Sherwin CMT (2023). The therapeutic effect of silymarin and silibinin on depression and anxiety disorders and possible mechanism in the brain: a systematic review. Cent Nerv Syst Agents Med Chem.

[36] Yi M, Manzoor M, Yang M, Zhang H, Wang L, Zhao L (2024). Silymarin targets the FXR protein through microbial metabolite 7-keto-deoxycholic acid to treat MASLD in obese mice. Phytomedicine.

[37] Piazzini V, D’Ambrosio M, Luceri C, Cinci L, Landucci E, Bilia AR (2019). Formulation of nanomicelles to improve the solubility and the oral absorption of silymarin. Molecules.

[38] Taleb A, Ahmad KA, Ihsan AU, Qu J, Lin N, Hezam K (2018). Antioxidant effects and mechanism of silymarin in oxidative stress induced cardiovascular diseases. Biomed Pharmacother.

[39] Dixit N, Baboota S, Kohli K, Ahmad S, Ali J (2007). Silymarin: A review of pharmacological aspects and bioavailability enhancement approaches. Indian J Pharmacol.

[40] Kshirsagar AD, Ingawale DK, Ashok P, Vyawahare NS (2009). Silymarin: A comprehensive review. Phcog Rev.

[41] Radko L, Cybulski WA (2007). Application of silymarin in human and animal medicine. J Pre-Clin Res.

[42] Tabari SA, Carpi S, Polini B, Nieri P, Esfahani ML, Moghadamnia AA (2019). Topical application of silymarin enhances cutaneous wound healing in rats. S Afr J Bot.

[43] Noori T, Sureda A, Shirooie S (2025). Ivermectin decreases inflammation and imiquimod–induced psoriasis-like skin lesions in rat via targeting TLR4/p65 NF-κB. Iran J Basic Med Sci.

[44] Guo Y, Wang S, Wang Y, Zhu T (2016). Silymarin improved diet-induced liver damage and insulin resistance by decreasing inflammation in mice. Pharm Biol.

[45] Stolf AM, Cardoso CC, Acco A (2017). Effects of silymarin on diabetes mellitus complications: A review. Phytother Res.

[46] Schäfer I, Hacker J, Rustenbach SJ, Radtke M, Franzke N, Augustin M (2010). Concordance of the psoriasis area and severity index (PASI) and patient-reported outcomes in psoriasis treatment. Eur J Dermatol.

[47] Okasha EF, Bayomy NA, Abdelaziz EZ (2018). Effect of topical application of black seed oil on imiquimod-induced psoriasis-like lesions in the thin skin of adult male albino rats. The Anat Rec.

[49] Li TY, Liang WL, Zhao YM, Chen WD, Zhu HX, Duan YY (2024). Alpha-Pinene-encapsulated lipid nanoparticles diminished inflammatory responses in THP-1 cells and imiquimod-induced psoriasis-like skin injury and splenomegaly in mice. Front Immunol.

[50] Li XP, Liu P, Li YF, Zhang GL, Zeng DS, Liu DL (2019). LPS induces activation of the TLR4 pathway in fibroblasts and promotes skin scar formation through collagen I and TGF-β in skin lesions. Int J Clin Exp Pathol.

[51] Yao L, Kan EM, Lu J, Hao A, Dheen ST, Kaur C (2013). Toll-like receptor 4 mediates microglial activation and production of inflammatory mediators in neonatal rat brain following hypoxia: Role of TLR4 in hypoxic microglia. J Neuroinflammation.

[52] Katiyar SK (2005). Silymarin and skin cancer prevention: Anti-inflammatory, antioxidant and immunomodulatory effects (Review). Int J Oncol.

[53] Esmaeil N, Anaraki SB, Gharagozloo M, Moayedi B (2017). Silymarin impacts on immune system as an immunomodulator: One key for many locks. Int Immunopharmacol.

[54] Weinstein GD, McCullough JL, Ross PA (1985). Cell kinetic basis for pathophysiology of psoriasis. J Invest Dermatol.

[55] Singh RP, Agarwal R (2002). Flavonoid antioxidant silymarin and skin cancer. Antioxid Redox Signal.

[56] Goldminz AM, Au SC, Kim N, Gottlieb AB, Lizzul PF (2013). NF-κB: an essential transcription factor in psoriasis. J Dermatol Sci.

[57] Al-Rasheed NM, Fadda LM, Al-Rasheed NM, Ali HM, Yacoub HI (2016). Down-regulation of NFkB, Bax,TGF-β, Smad-2mRNA expression in the livers of carbon tetrachloride treated rats using different natural antioxidants. Braz Arch Biol Technol.

[58] Altindağ F (2022). Silymarin ameliorates cisplatin-induced nephrotoxicity by downregulating TNF-α and NF-kB and by upregulating IL-10. J Exp Clin Med.

[59] He X, Wei Z, Zhou E, Chen L, Kou J, Wang J (2015). Baicalein attenuates inflammatory responses by suppressing TLR4 mediated NF-κB and MAPK signaling pathways in LPS-induced mastitis in mice. Int Immunopharmacol.

[60] Linh NTT, Giang NH, Lien NTK, Trang BK, Trang DT, Ngoc NT (2022). Association of PSORS1C3, CARD14 and TLR4 genotypes and haplotypes with psoriasis susceptibility. Genet Mol Biol.

[61] Saleh HA, Yousef MH, Abdelnaser A (2021). The anti-inflammatory properties of phytochemicals and their effects on epigenetic mechanisms involved in TLR4/NF-κB-mediated inflammation. Front Immunol.

